# TB Risk Perceptions among Medical Residents at a Tertiary Care Center in India

**DOI:** 10.1155/2017/7514817

**Published:** 2017-11-22

**Authors:** Geeta S. Pardeshi, Dileep Kadam, Ajay Chandanwale, Andrea Deluca, Pranali Khobragade, Malan Parande, Nishi Suryavanshi, Aarti Kinikar, Anita Basavaraj, Sunita Girish, Sangita Shelke, Nikhil Gupte, Jason Farley, Robert Bollinger

**Affiliations:** ^1^Department of Community Medicine, Vardhman Mahavir Medical College and Safdarjung Hospital, New Delhi, India; ^2^Byramjee Jeejeebhoy Government Medical College/Sassoon General Hospital, Pune, Maharashtra, India; ^3^Department of International Health, Johns Hopkins Bloomberg School of Public Health, Baltimore, MD, USA; ^4^Byramjee Jeejeebhoy Government Medical College/Johns Hopkins Clinical Trials Unit, Pune, Maharashtra, India; ^5^School of Nursing, Johns Hopkins University, Baltimore, MD, USA; ^6^Division of Infectious Diseases and International Health, Johns Hopkins University School of Medicine, Baltimore, MD, USA

## Abstract

**Setting:**

Government tertiary health care center in India.

**Objective:**

To understand the perceptions of medical residents about their risk of developing TB in the workplace.

**Design:**

Cross-sectional study in which a semistructured questionnaire which included an open-ended question to assess their main concerns regarding TB in workplace was used to collect data.

**Results:**

Out of 305 resident doctors approached, 263 (94%) completed a structured questionnaire and 200 of these responded to an open-ended question. Daily exposure to TB was reported by 141 (64%) residents, 13 (5%) reported a prior history of TB, and 175 (69%) respondents were aware of TB infection control guidelines. A majority reported concerns about acquiring TB (78%) and drug-resistant TB (88%). The key themes identified were concerns about developing drug-resistant TB (*n* = 100; 50%); disease and its clinical consequences (*n* = 39; 20%); social and professional consequences (*n* = 37; 19%); exposure to TB patients (*n* = 32; 16%); poor infection control measures (*n* = 27; 14%); and high workload and its health consequences (*n* = 16; 8%).

**Conclusion:**

Though many resident doctors were aware of TB infection control guidelines, only few expressed concern about lack of TB infection control measures. Doctors need to be convinced of the importance of these measures which should be implemented urgently.

## 1. Introduction 

Health Care Workers (HCWs) are known to be at risk of acquiring tuberculosis in the workplace [1]. In response to this risk, the World Health Organization published guidelines for prevention of TB in health care facilities and policy on TB infection control [2, 3]. In India, studies have also reported an increased risk of TB among HCWs [4–8]. The government has issued airborne infection control recommendations for health care settings [9]. However, due to structural, administrative, and behavioral factors [10–13], implementation of these guidelines has been poor [14–17].

Recently a study estimated TB incidence of 3,279 per 100,000 person years among resident doctors in a tertiary care center in India [18]. In our study at the same institute which was conducted to assess the resident doctors' attitudes toward TB patients, only 49% reported feeling compassion for and desire to help TB patients. The remaining 51% reported either feeling compassion yet avoiding TB pateints, having a fear and thinking that they may cause infection, or having no particular feeling [19].

These results raise concerns about the ability of the residents to follow the TB prevention guidelines and its impact on their attitudes towards TB patients. Health care workers are key stakeholders for interventions to prevent occupationally acquired TB. Understanding the resident doctors TB risk perceptions will aid in implementing effective interventions for prevention of occupationally acquired TB at institutional level.

## 2. Methods

### 2.1. Study Design and Setting

A cross-sectional study was conducted at B.J. Government Medical College and Sassoon General Hospital, a large, public tertiary health care center in Pune, India. Sassoon General Hospital is a government medical college hospital which has total bed strength of 1,296 and an average OPD (Out-Patient Department) attendance of 45,000 per month.

### 2.2. Study Population

The study participants were postgraduate medical resident doctors, assigned to clinical and laboratory departments. A total of 325 residents were eligible for study participation in September 2014, when the data were collected.

### 2.3. Tool

A semiquantitative questionnaire was administered to consenting residents. The questionnaire was finalized after pretesting in a pilot study. It included questions on sociodemographic variables, duration of work, exposure to TB patients/specimens, past history of TB, and knowing a resident who had TB. Two questions assessed whether the residents were concerned about TB and drug-resistant TB at workplace. The knowledge and awareness of the resident doctors about the administrative measures, environmental measures, and use of respiratory protective devices for prevention of occupationally acquired TB in health care settings was assessed using a Likert scale (strongly disagree, disagree, no opinion, agree, strongly agree). In addition, the questionnaire included one open-ended question asking residents to document their main concerns regarding development of TB at workplace. All questions and answers were in English, the same language as their medical training.

### 2.4. Recruitment

The study investigators attended regular departmental meetings, where they explained the research project to the residents and invited them to participate in the study. The consenting residents were given a self-administered anonymous questionnaire.

### 2.5. Ethical Review

The study protocol was approved by the ethics committees of B.J. Government Medical College and the Johns Hopkins University School of Medicine. Written informed consent was obtained from each respondent.

### 2.6. Analysis

Descriptive analysis was done using proportions, mean, and standard deviation. For analyzing the responses on the Likert scale, responses “strongly disagree, disagree, and no opinion” were combined into one group and “strongly agree and agree” were combined into another group. The responses to the open-ended question were analyzed manually in an excel sheet, using thematic content analysis [20]. Two investigators developed a coding scheme for all the responses and applied it to the dataset. An interrater reliability analysis for the two sets of codes was done using Kappa statistic. Codes were transformed into subthemes, which were sorted into key themes by identifying similar patterns and commonalities. Exemplary quotations were identified for each theme. The frequency of respondents reporting each key theme was calculated.

## 3. Results

Out of the 325 residents registered for postgraduate courses at the institute, 305 (94%) could be contacted. Of them 263 (86%) responded to questions assessing knowledge and 200 (76%) responded to the open-ended question about their concerns for contracting TB in the workplace. The characteristics of residents who responded only to the structured questions and to both the structured and open-ended questions are described in Table 1. Overall, majority of the respondents were male (60%) and had daily exposure to a known TB patient or specimen (64%).

Thirteen (5%) residents reported that they were previously diagnosed and treated for TB and 40 (16%) had been assessed for TB based on symptoms during their period of medical education. Most residents were concerned about being infected with TB at the workplace (78%) and an even greater proportion of residents (88%) expressed specific concern about acquiring drug-resistant TB in the workplace.

Table 2 describes the knowledge of the resident medical doctors regarding measures for prevention of occupationally acquired TB. A majority of the respondents had knowledge about administrative measures like early detection and separation of patients and cough etiquette in patients as well as environmental measures like good ventilation. Only 175 (69%) of resident doctors were aware of any guidelines for prevention of transmission of TB in health care settings. Many residents, that is, 145 (56%), were not aware of the use of ultraviolet (UV) gamma radiation for reducing the risk of transmission of TB in health care setting. While 166 (64%) respondents knew that N 95 masks reduce the risk of acquiring TB only 96 (37%) were aware about the need for fit testing.

To further understand the residents' main concerns about acquiring TB in the workplace, the 200 responses to the open-ended question were coded and analyzed. The level of agreement between the coders was high, with a Kappa of 0.85 (*p* < 0.001, 95% CI: 0.83–0.92). Six key themes were identified. Developing drug-resistant TB was the most common concern reported by 100 (50%) residents. The second most common concern, expressed by 39 (20%) residents, was about developing TB disease and its clinical consequences. Concerns about social and professional consequences of developing TB were reported by 37 (19%) residents. Other concerns expressed included exposure to TB patients at the workplace (*n* = 32; 16%); poor TB infection control measures (*n* = 27; 14%); and high workload and its health consequences (*n* = 16; 13%).

The six key themes, their subthemes, and representative comments from the responses to open-ended question are as follows.

### 3.1. Concern about Developing Drug-Resistant TB (DR-TB) in the Workplace

Concern about developing drug-resistant TB in the workplace was the most common key theme reported by 100 residents who responded to the open-ended question. Subthemes from these responses included concerns about side effects of DR-TB treatment, delayed diagnosis of DR-TB, and poor treatment outcomes due to DR-TB.

Representative responses about this theme and subthemes included the following:

*“I am worried about the risk of MDR/XDR TB, as the treatment is very difficult to follow because of side effects.”* (Age 27, Female, Ophthalmology)

*“*…*worried about getting diagnosed with MDR/XDR TB. I would prefer to be screened directly for MDR TB and start treatment accordingly to prevent delays.”* (Age 26, Male, Surgery)

*“About MDR TB, there will be no definitive line of management; because I have seen Category IV patients go in relapse/failure.”* (Age 27, Male, Orthopedics)

### 3.2. Concern about Developing TB Disease and Clinical Consequences of TB

Concern about developing TB in the workplace and its clinical consequences was the second most common key theme reported by the residents in response to the open-ended question. Subthemes from these responses included concerns about type of TB, clinical complications of TB, adverse effects of treatment, the long duration of treatment, and potential for hospitalization.

The following quotes represent this theme and subthemes:

*“It can affect body system in the form of extra-pulmonary tuberculosis which remains undiagnosed for much time.”* (Age 26, Female, Medicine)

*“It could affect fertility.”* (Age 26, Female, Pediatrics)

*“The duration of treatment is lengthy.”* (Age 27, Male, Medicine)

*“I am worried about the adverse effects of drugs.”* (Age 26, Female, Ophthalmology)

*“Worried about the stay in TB ward.”* (Age 30, Male, Psychiatry)

### 3.3. Concern about the Social and Professional Consequences of Developing TB

The residents expressed concern about a number of social and professional consequences of developing TB. The subthemes included academic loss, transmitting the infection to family members, and social stigma attached to the disease.

Responses representing this theme were as follows:

*“I am worried about absenteeism from work, gap in residency.”* (Age 26, Female, Medicine)

* “Worried about transmitting TB to others.”* (Age 26, Male, Preventive and Social Medicine)

*“My colleagues will avoid staying with me if I develop TB.”* (Age 28, Female, Medicine)

### 3.4. Concern about Exposure to TB Patients in the Workplace

This key theme included subthemes such as concerns about frequent encounters with TB patients, with undiagnosed and undisclosed TB patients, and patients with poor cough etiquettes. The residents from different departments expressed concern about specific types of exposures. For example, the resident doctors from the ophthalmology department perceived certain procedures, which they performed from close quarters, to increase the risk of infection.

Representative responses about this theme and subthemes included the following:

*“There are many Koch's patients in the hospital campus. There is significant direct exposure to TB patients in OPD.”* (Age 28, Male, Community Medicine)

*“Many patients in the OPD have undiagnosed TB. Hence there is more risk.”* (Age 26, Male, Surgery)

*“Some patients hide their TB status from doctors.”* (Age 25, Female, Otorhinolaryngology)

*“Some patients cough right in front of our face in OPDs.”* (Age 25, Female, Dermatology)

*“When patients with TB and co-morbidity such as hypertension, diabetes mellitus are admitted in the wards a call is sent to ophthalmology department. We have to examine such patients with direct ophthalmoscope from close quarters. Other procedures like sac syringing, slit lamp examination also pose a risk of TB transmission.”* (Age 28, Female, Ophthalmology)

### 3.5. Concern about Poor Infection Control Measures

There were multiple concerns expressed about poor infection control measures. The subthemes included inadequate patient screening for TB, lack of room ventilation, nonfunctional mechanical ventilation, and lack of N-95 masks. The resident doctors were concerned with both the natural and mechanical ventilation in general and specifically in the laboratories and enclosed rooms of radiology department. Only ten residents reported concern about lack of N-95 masks at workplace. Residents highlighted the importance of ventilation in enclosed rooms as well as practical difficulties and even a negligent attitude towards the use of protective equipment.

A few responses under this theme were as follows:

*“*…*inadequate testing and screening for TB.”* (Age 25, Male, Anesthesia)

*“Being doctors we are exposed to burden of diverse (MDR) flora and still we have to work without necessary equipment (N 95 masks)”* (Age 28, Male, Pulmonary Medicine)

*“We have a risk during certain procedures like USG. All our investigations are conducted in enclosed dark rooms with mechanical ventilation. If this fails and is not repaired, our risk increases.”* (Age 26, Male, Radiology)

*“*…*it is hardly possible for me to use the mask (N95) all the time. It causes suffocation and problems in communication.”* (Age 26, Male, Ophthalmology)

*“We do not use N 95 masks every time we do bronchoscopy. Use it only if we suspect the patient has TB*…*This is mere negligence.”* (Age 29, Male, Pulmonary Medicine)

### 3.6. Concern about High Workload and Its Health Consequences

The subthemes under this key theme were concerns about excessive workload, long working hours, improper nutrition, and inadequate rest among the residents.

The following responses represent this theme:

*“With odd working hours, sleep and food schedule we are at risk of developing TB”* (Age 26, Male, Anesthesia)

*“Because of excessive physical strain and mental fatigue residents with heavy postings are not able to follow standard practices even if they know about them and then they fall for infection”*. (Age 25, Male, Medicine)

*“There is excessive workload, especially in the first year. Need to redistribute the work among all residents*…*”* (Age 28, Male, Pediatrics)

The key themes and subthemes which emerged from the study are summarized in Table 3.

## 4. Discussion

The study was conducted in a medical college hospital without a separate institutional TB infection control policy, in which residents have a heavy workload and frequent encounters with TB patients. The results of our study show that most medical residents in this public teaching hospital in India perceive TB as an occupational hazard, and many expressed concern about the consequences of developing TB disease. The incidence of TB among colleagues and the fact that many of the interventions for prevention of TB in health care settings need to be initiated at the institutional level rather than at an individual level could have contributed to these risk perceptions. There is a need to train the resident doctors as well as generate and highlight evidence demonstrating feasibility and effectiveness of the interventions for prevention of TB within health care settings in the Indian scenario. This may convince them of the need and also generate a demand for implementation of the TB infection control measures.

A majority of the resident doctors know about the role of administrative measures and ventilation in prevention of occupationally acquired TB. Less than 70% of them are aware of any guidelines for prevention of TB in health care settings, use of ultraviolet radiation and N 95 masks for prevention of occupationally acquired TB. Few were aware about the need of fit testing prior to using N 95 masks. In addition, only 39% of the residents reported to have been adequately trained in TB infection control measures. These gaps in knowledge indicate the need to incorporate the topic of prevention of occupationally acquired TB in their training program. The lack of knowledge about use of N 95 masks could also be due to either nonavailability or nonuse of the masks at workplace.

Responses to the open-ended questions indicate that their concerns go beyond the concerns about measures for preventing TB infection at workplace. These include concerns related to clinical, social, and professional implications of getting the disease and excessive workload.

A major concern is getting TB disease, including acquiring drug-resistant TB. Many of the resident doctors know of a colleague who has been diagnosed with TB. Having come across patients with TB and knowing another resident with TB reinforces this perception. A study conducted in this institute has reported that medical trainees in India are at risk of TB, including MDR TB [18]. Fear of death, duration, and side effects of the treatment regimens and hospitalization have been reported in this study. A study has reported similar fears among HCWs working in MDR/XDR TB wards in South Africa [21]. The perceptions of stigma about the disease may be influenced by the sociocultural milieu in the community. Being students, the resident doctors are also concerned about the anticipated extension of academic period resulting from the disease.

The resident doctors mention workload and its impact on their diet and resting hours. Concerns related to excessive workload and inadequate sleep and rest time leading to stress, especially in the first year of residency have been described in other studies too [22–25]. The hospital has a hierarchy system in which the major burden of work falls on the junior residents. Redistributing duties so that work hours are more evenly distributed among the health care workers will address some of the issues related to excessive workload. In certain procedures, the doctors examine the patients from close quarters, which is perceived to increase risk of TB transmission. Respondents in this study have mentioned some of these procedures, direct ophthalmoscopy, sac syringing, and slit lamp examination. Standard operating procedures should be established for trainees who perform them. TB wards, ART centers, bronchoscopy rooms, Intensive Care Units, radiology department, autopsy suites, and TB laboratories are designated high risk areas [9]. Good ventilation, masks for patients, and appropriate personal protective equipment for the doctors should be ensured for high risk procedures and areas. Using N-95 masks has been shown to lower the risk of incident LTBI [26]. However, the study respondents have reported practical problems in wearing masks for prolonged periods of time, as needed in high prevalence settings. Other studies have reported association of discomfort and headache with prolonged use of masks [27, 28]. The institute should formulate a policy for using N-95 masks and motivate and monitor its use among the residents. Addressing the resident doctors' concerns will not only facilitate prioritization of the implementation of interventions for prevention of TB transmission but also ensure compliance to the measures when they are implemented.

A majority of the medical resident doctors were concerned about TB disease, especially drug-resistant TB. Though many of the resident doctors were aware of TB infection control guidelines only few of them expressed concern about exposure to TB patients and lack of TB infection control measures at the institutional level. There is a need to train the resident doctors and highlight specific evidence demonstrating the feasibility and efficacy of these guidelines in prevention of TB in Indian scenario. Simultaneously, the health care system has to fulfill the reciprocal obligation of implementing these guidelines.

## Figures and Tables

**Table 1:** Characteristics of medical residents responding to TB risk questionnaire.

Variables	Responded to only structured questions	Responded to structured and open-ended questions^*∗*^
Study participants: *N*	263	200
Mean age: years (SD)	27.2 (3.6)	27.3 (3.6)
Male gender: *N*/responses (%)	147/263 (60)	111/200 (56)
Year of residency: *N*/responses (%)		
First year	97/263 (37)	73/200 (36)
Second year	87/263 (33)	66/200 (33)
Third year	79/263 (30)	61/200 (31)
Working ≥12 hours/day: *N*/responses (%)	115/251 (46)	94/194 (49)
Daily exposure to known TB patients or specimens: *N*/responses (%)	141/222 (64)	104/168 (62)
Assessed for TB in the past: *N*/responses (%)	40/254 (16)	35/193 (18)
Past history of TB: *N*/responses (%)	13/253 (5)	12/193 (6)
Know a resident colleague with TB: *N*/responses (%)	115/251 (46)	94/194 (49)
Concerned about being infected with TB at workplace: *N*/responses (%)	205/263 (78)	161/200 (81)
Concerned about being infected with drug-resistant TB at workplace: *N*/responses (%)	232/263 (88)	183/200 (92)

^*∗*^Subset of residents who responded to the open-ended question about TB risk concerns.

**Table 2:** Knowledge about measures to prevent occupationally acquired tuberculosis amongst medical resident doctors.

Measures	Responses *N* (%)	
	Agree	Disagree/ No opinion
*Administrative measures*		
Early detection and separation of patients with TB will reduce the risk of TB for HCWs (*N* = 261)	229 (88)	32 (12)
Cough etiquette in patients will reduce the risk of TB in HCWs (*N* = 257)	230 (89)	27 (11)
There are guidelines for prevention of transmission of TB in Health Care Set Up (*N* = 252)	175 (69)	77 (31)
*Environmental measures*		
Good ventilation at workplace will reduce the risk of TB in HCWs (*N* = 261)	238 (91)	23 (9)
Ultraviolet gamma irradiation can be used to reduce risk of transmission of TB at workplace (*N* = 257)	112 (44)	145 (56)
*Respiratory protective devices*		
HCWs can reduce their risk of acquiring TB by wearing N 95 masks (*N* = 259)	166 (64)	93 (36)
Fit testing is needed before using N 95 masks (*N* = 259)	96 (37)	163 (63)

**Table 3:** TB risk perceptions among resident doctors, key themes and subthemes.

Key themes	Subthemes
Developing drug-resistant TB (*n* = 100)	Side effects of DR-TB treatment; delayed diagnosis of DR-TB; poor treatment outcomes
Disease and its clinical consequences (*n* = 39)	Type of TB; clinical complications of TB; adverse effects of treatment; long duration of treatment; potential for hospitalization
Social and professional consequences (*n* = 37)	Academic loss; transmitting the infection to family members; social stigma attached to the disease
Exposure to TB patients/specimens (*n* = 32)	Frequent encounters with TB patients; encounters with undiagnosed cases; encounters with undisclosed TB patients; patients with poor cough etiquettes
Poor infection control measures (*n* = 27)	Inadequate patient screening for TB; Lack of room ventilation; Nonfunctional mechanical ventilation; Nonavailability and nonuse of N-95 masks
High workload and its health consequences (*n* = 16)	Workload; working hours; improper nutrition; inadequate rest
